# Antibiotic stewardship and nosocomial infection prevention in critically ill patients: a quality improvement program

**DOI:** 10.1590/1806-9282.20231282

**Published:** 2024-05-20

**Authors:** Nayá Saad Custódio, Luana Fernandes Machado, Graziela Denardin Luckemeyer, Juliana Devós Syrio, Isabela Shumaher Frutuoso, Debora Augusto Valverde Chanes, Luciana Tirelli Kaltenbacher, Melissa Maia Braz, Mara Correa Lelles Nogueira, Joelma Villafanha Gandolfi, Suzana Margareth Lobo

**Affiliations:** 1 Faculty of Medicine of São José do Rio Preto – São José do Rio Preto (SP), Brazil.; 2 Hospital de Base de São José do Rio Preto, Faculty of Medicine of São José do Rio Preto, Intensive Care Unit – São José do Rio Preto (SP), Brazil.; 3 Comissão de Controle de Infecção Hospitalar, Hospital de Base de São José do Rio Preto, Faculdade de Medicina de São José do Rio Preto – São José do Rio Preto (SP), Brazil.

**Keywords:** Antibiotic stewardship, Nosocomial infection, Nosocomial pneumonia, Quality improvement, Procalcitonin

## Abstract

**OBJECTIVE:**

The objective of this study was to evaluate the impact of the implementation of a bundle of interventions through a "Program for Antibiotic Management and Nosocomial Infection Prevention" in the intensive care unit on antibiotic and devices use and healthcare-associated infections.

**METHODS:**

This was a quasi-experimental study of consecutive series of cases in periods before and after the establishment of protocols and checklists for the use of antibiotics as well as other measures to prevent healthcare-associated infection as part of a quality improvement program. Antimicrobial consumption was assessed by the defined daily dose.

**RESULTS:**

A total of 1,056 and 1,323 admissions in the pre-intervention and post-intervention phases, respectively, were evaluated. The defined daily dose per 100 patient-day decreased from 89±8 to 77±11 (p=0.100), with a decrease in carbapenems, glycopeptides, polymyxins, penicillins, and cephalosporins. The rates of ventilator and central venous catheter use decreased from 52.8 to 44.1% and from 76 to 70%, respectively. The rates of healthcare-associated infection decreased from 19.2 to 15.5%.

**CONCLUSION:**

Quality improvement actions focused primarily on antimicrobial management and prevention of healthcare-associated infection are feasible and have the potential to decrease antibiotic use and healthcare-associated infection rates.

## INTRODUCTION

Infections caused by multidrug-resistant pathogens (MDRPs) are a growing problem in intensive care units (ICUs) due to the complexity of patients, high consumption of antimicrobials, and inappropriate use of these drugs^1,2^. In the study "Extended Study on Prevalence of Infection in Intensive Care III" (EPIC III) with 15,165 patients admitted to the ICU, 54% of patients had at least one suspected infection, 70% received at least one ATB, and the mortality rate was 30%^3^. The most notable finding of EPIC III was how little has changed in terms of the prevalence of infection and associated mortality over three decades^4^.

Important steps for more rational use of antibiotics (ATBs) are the rapid, adequate, and optimized initiation of empirical treatment, use of therapeutic regimens that allow maximum bactericidal effect, with rapid reduction of bacterial load, and early de-escalation and discontinuation of antimicrobial therapy. According to the guidelines of the Survival Sepsis Campaign (SSC), de-escalation or interruption of treatment should be performed as soon as possible^5^. The SSC expert panel also suggested the use of procalcitonin (PCT) along with clinical evaluation to decide when to discontinue antimicrobials.

Antimicrobial Stewardship Programs can lead to significant reductions in ATB use and in the costs in healthcare facilities^6^. In addition, the use of protocols and checklists has been shown to be effective in improving processes and outcomes, including the reduction of nosocomial infections^7,8^. Programs to reduce ICU-acquired infections ("Zero Bacteremia" and "Zero ventilator-associated pneumonia") in more than 200 ICUs in Spain led to a significant reduction in infection rates and ATB use in the participating units^9,10^. The aim of this study was to evaluate the impact of a set of actions focused on ATB management and infection prevention in ICUs on ATB use and healthcare-associated infections (HAIs), particularly respiratory tract infections.

## METHODS

This quasi-experimental study was conducted to evaluate the impact of a multifaceted intervention ("Program for Antibiotic Management and Nosocomial Infection Prevention") in the Intensive Care Center of a tertiary teaching hospital (2 units with 40 clinical-surgical ICU beds). The study was approved by the Ethics Committee (CAAE: 12539119.4.0000.5415). Data on the rates of ATB, device use, and HAI of all patients hospitalized between August 1 and December 31, 2018 (post-intervention group) were recorded by a trained team. The same data were retrieved from the records (pre-intervention group) which consisted of patients hospitalized between August 1 and December 31, 2017. The primary objective of the study was to evaluate the impact of the implementation of this program on ATB and device use. The secondary objective was to evaluate the impact of the program on the occurrence of HAI.

**Figure 1:**
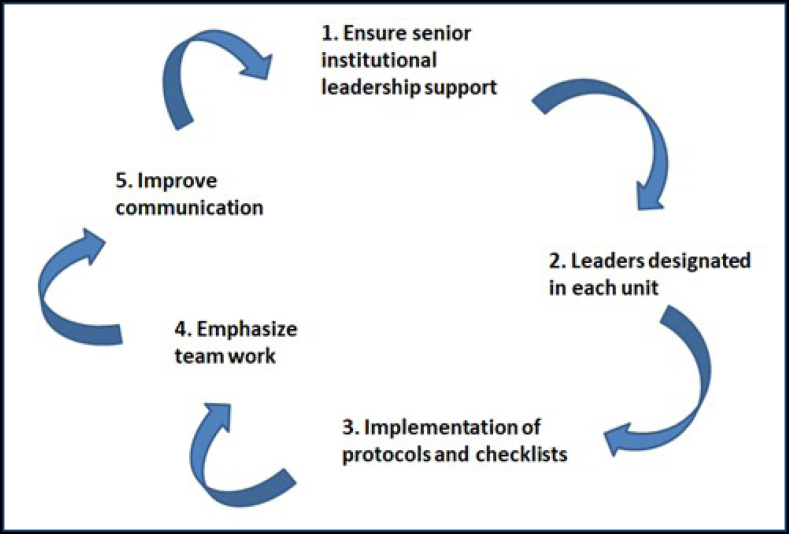
Main steps of the antibiotic stewardship and nosocomial infection prevention program.

The rising rate of HAIs was considered critical in the analysis of the study team in the first half of 2018 (from January 1 to July 31). During this period, monthly meetings were held to discuss and implement the "Program for Antibiotic Stewardship and Nosocomial Infection Prevention" in the ICU. The Program consisted of a set of sequential interventions that included: setting up a working group, analysis of ICU problems using the Ishikawa diagram, prioritization of problems using the gravity, urgency, and tendency (GUT) matrix, use of the Plan, Do, Check and Adjust (PDCA) method, as well as training and establishment of protocols and checklists^11^. The following actions were deemed as priorities and implemented by the working group (Figure 1):

1. We ensured the support of senior institutional leadership and appointed leaders for the working group for each ICU (intensive care and infection control physicians, nurses, and pharmacists) to ensure adherence to the program. The complete team was composed of four intensivists (SML, CFM, GBL, JDS, and MRRGJ), one infectologist (MMB), three nurses (IFS, DAVC, and LTK), two microbiologists (MCLN and MTGA), one clinical pathologist (MGLO), two pharmacists (JVG and HTAO), and one internist medical student (NSC).
2. Protocols were established and trained for the use of ATB considering dosage (loading and maintenance), prolonged infusion time for beta-lactams and carbapenems, duration of treatment, as well as recommendations for the use of combined therapy in specific cases.
3. A checklist to identify patients at high risk for MDRP infections was implemented, in addition to actively monitoring colonization with weekly rectal swab cultures.
4. Guidelines for de-escalation and termination of treatments guided by clinical response, cultures, and daily PCT were determined^12,13^. PCT values were used as a recommendation for the discontinuation of ATB in the post-intervention phase, with discontinuation strongly encouraged if PCT ≤0.25 μg/l and encouraged if there was a decrease of ≥80% of the peak level or ≥0.25 and ≤0.5 μg/l.
5. The measures to prevent HAI consisted in reviewing of the protocols and training of the team on hand hygiene, cleaning of the rooms and equipment, and bathing and oral hygiene of patients, as well as daily assessment of the possibility of removing catheters, probes, and drains. Adherence rates were analyzed through checklists of these measures with periodic disclosures of HAI rates through meetings and dashboard use.

Antimicrobial use was evaluated by the defined daily dose (DDD), which is the average daily maintenance dose of the antimicrobial (in grams) taken by a 70-kg adult for the main therapeutic indication of that drug. The values of the total amount of the drugs taken were obtained through the drug dispensing system of the hospital’s pharmacy, considering the antimicrobials used by all patients during ICU stay. The formula for calculating the DDD was $\frac{A\times 100}{B\times P}$ , where *A* is the total use of the antimicrobial (in grams), in the period of time considered, *B* is the standard daily dose of the antimicrobial (in grams) calculated for 70-kg adults without renal failure [defined by the World Health Organization (WHO)], and p is patient-day, in the period of time considered^14^.

The indicators of device use and HAI were provided by the Hospital Infection Control Commission (CCIH) of the institution for the two study periods. The diagnostic criteria follow those set by the Centers for Disease Control and Prevention (CDC).

### Statistical analysis

This was a pragmatic study with a convenience sample whose size calculation was not performed. All data were analyzed using the Statistical Package for the Social Sciences (SPSS) software. The means and variances of the phases were evaluated by Student’s t-test or the Mann-Whitney test, depending on the distribution of the data. The frequencies of the qualitative and categorical variables of the subgroups were assessed using the chi-square test or Fisher’s exact test. Statistical significance was set at p<0.05. The relative risks (RR) as the ratio of risk of an event were calculated in both groups.

## RESULTS

In the first period of evaluation (pre-intervention) 1,056 patients were admitted to ICUs. In the second period (post-intervention) 1,323 patients were admitted. ICU and hospital stay decreased from 7.3±7.4 to 5.9±6.7 days and from 16.7±15.5 to 13.0±12.6 days (p<0.05 for both), respectively (Table 1). The hospital standardized mortality ratio (SMR) was 1.28 in the pre-phase and 1.14 in the post-phase.

**Table 1:** Epidemiological and clinical profile of patients hospitalized in the pre- and post-intervention phases of the study.

	Pre-intervention	Post-intervention
Number of patients	1,056	1,323
Clinic (%)	48.7	54.6
Elective surgery (%)	18.2	20.4
Emergency surgery (%)	33.1	22.5
Age (years)	54±19	59±18
Sex male/female (%)	59/41	55/45
SAPS 3	54.6±22	51.5±21
Charlson comorbidity index	1.5±2.0	1.5±1.9
ICU occupancy rate (%)	88.4	88.3
Readmission rate < 48 h (%)	2.6	1.6
SMR (hospital)	1.28	1.14
ICU stay (days)	7.3±7.4	5.9±6.7*
Length of hospital stay (days)	16.7±15.5	13.0±12.6*
Death ICU (%)	28.4	24.6*

M/F: male/female; SAPS 3: simplified acute physiology score 3; ICU: intensive care unit; SMR: standardized mortality rate.

* p<0.05 vs. pre-intervention.

The DDD per 100 PD decreased from 89±8 to 77±11 (p=0.100). The reduction was significant in the cephalosporin group (15.4±3.8 DDD per 100 PD to 10.5±1.4 DDD per 100 PD; p=0.045) (Table 2). There was a decreasing trend in all groups, especially for polymyxins (p=0.07), with the exception of aminoglycosides for which the trend was to increase, although they were less used in both periods.

**Table 2:** Consumption of the main classes of antibiotics according to the defined daily dose/100 patient-day in the pre- and post-intervention phases of the study.

	Pre-intervention	Post-intervention	95%CI	p-value
Carbapenems	29.3±4.8	27.8±3.9	-5.05; 8.07	0.603
Glycopeptides	18.4±.3	17.5±3.0	-2.79; 4.64	0.549
Polymyxins	14.3±2.4	10.8±2.9	-0.52; 7.45	0.079
Penicillins	7.6±2.2	5.4±1.3	-0.65; 4.99	0.109
Cephalosporins	15.4±3.8	10.5±1.4	0.16; 9.60	0.045
Aminoglycosides*	4.1±1.6	5.3±2.0	-3.90; 1.50	0.335
All	89.0±8.5	77.3±11.0	-2.90; 26.50	0.100

Results are presented as mean ± standard deviation. Student’s t-test was used for differences.

* Combined therapy with the addition of an aminoglycoside (amikacin and gentamicin; 3 days) was recommended by the protocol for patients at high risk of MDRP, septic shock, and neutropenia (2,5), and this led to a slight increase in the use of this class of ATB.

The rates of ventilator and central venous catheter use fell from 52.8 to 44.1% (RR 0.86, 95%CI 0.79–0.93, p<0.005) and from 76 to 70% (RR 0.92, 95%CI 0.87–0.97, p<0.001), respectively (Table 3). The rates of HAI decreased from 19.2% in the pre-intervention phase to 15.5% (RR 0.81, 95%CI 0.67–0.97, p<0.005) in the post-intervention phase and the incidence density of VAP per 1,000 MV-day decreased from 20.3 to 14.3%. Clinical incidence density for blood stream infections (BSI/1000 CC-day) was zero for both periods.

**Table 3:** Indicators of the annual report of the Hospital Infection Control Commission in the pre- and post-intervention phases of the study.

Variable		Pre-intervention	Post-intervention	p-value
Devices				
	Mechanical ventilation-day	3,829	3,489	0.034
	Central venous catheter-day	5,504	5,577	0.183
	Use of central venous catheter (%)	75.8	70.5	0.000
Healthcare-associated infection				
	Number of patients with HAI/month	166	145	0.026
	HAI/patient	1.12	1.09	0.872
	Infection rate per 1,000 patient-day (%)	2.03	1.83	0.088
	Patients with pneumonia	105	61	0.000
	Incidence density VAP/1,000 MV-day (%)	20.3	14.3	0.037

Data are presented as numbers or (%) when indicated. Student’s t-test was used for differences. VAP incidence density is the number of ventilator-associated pneumonias (VAP) in the month/number of ventilator-day in the month × 1,000. Incidence density of bloodstream infection (BSI) is BSI month/number of central catheter-day at month × 1,000 (clinical diagnosis).

## DISCUSSION

After the implementation of the program, the DDD per 100 PD decreased from 85.0±8.0 to 72±9.6, and the reduction was more significant in the use of cephalosporin. Other authors have shown similar results. A quality improvement program in neonatal ICUs was successful in reducing the rate of ATB use through a rigorous ATB management education process^15^. A large study including 77 ICUs in nine Latin American countries evaluated the impact of an ATB management program on the adequacy of antimicrobial prescriptions and HAI^16^. The ICUs with ≥75th percentile in the final scores of adherence to the program had a greater reduction in the use of ATBs (143.4 vs. 159.4 DDD per 100 PD compared with the 25th percentile). In Saudi Arabia, an ATB management program conducted between 2015 and 2016 reduced the use of ATBs by 28.4%, which was a higher reduction than that obtained in our study (15%), but the observation time was longer^17^.

The use of biomarkers such as PCT or C-reactive protein has the potential to improve the therapeutic management of patients with infections and sepsis. In our ICUs, serum PCT measurement was previously in use to aid in the diagnosis of infection and sepsis in association with clinical judgment based on signs, symptoms, laboratory tests, cultures, and radiological exams in the phase PRE of the study. The use of PCT to discontinue antimicrobial therapy was only standardized after the intervention. It is possible that there was an impact of its use in the duration of ATB therapies; however, this study does not allow definitive conclusions of a cause–effect relationship due to its pragmatic design. Randomized controlled trials have suggested that the use of PCT to guide discontinuation of ATB therapy might decrease the use of ATBs by around 1 day^18-20^. The SSC 2021 expert panel suggested its use combined with clinical evaluation to decide when to discontinue antimicrobials based on data from 14 randomized controlled trials in patients with sepsis and from two studies that included critically ill patients in general^5^.

The use of daily checklists was important to reduce the use of devices and may have impacted the rate of HAI and therefore DDD. However, the pragmatic nature of this study does not allow us to determine the individual effect of these measures. In view of the complexity of the scenario in critically ill patients, multiple interventions are often complementary and necessary, as it is more likely that using bundles, a set of interventions rather than isolated interventions works better. In addition, it has been demonstrated by other authors that even simple education programs can lead to optimization in the use of ATBs^18^.

The main strength of this study is the careful planning, execution, and evaluation of an integral patient safety and educational program involving wide technical support from a multidisciplinary team. However, we should highlight some limitations. The results achieved were possibly related to the program implemented. However, the pragmatic nature of before-after studies with such a design (non-random allocation) does not allow us to state that these results were definitely due to the intervention as other variables could not be controlled and is rather hypothesis generating and should be confirmed in large randomized trials. Nevertheless, before-and-after studies are feasible and can be used as pilots for future randomized studies, and it has been demonstrated by other authors that even simple education programs can lead to optimization in the use of ATBs^18^. Using total daily dose (TDD) could have been a more reliable to access AB use. However, DDD is often used to assess trends in the use of these drugs and make comparisons between population groups. Finally, it may not be possible to generalize the findings of this study, but we believe that they could be applied to large tertiary or university hospitals where the consumption of carbapenems, polymyxins, and glycopeptides is high in the ICUs.

Antimicrobial resistance is a global priority. Currently, with the overuse of ATBs exacerbated by the COVID-19 pandemic, combating antimicrobial resistance is an even greater challenge, and initiatives to optimize the proper use of ATBs are urgent. Whether programs like this can effectively lead to more restricted use of ATBs and reduction in rates of ATB resistance and costs should be the subject of further multicenter randomized studies.

In conclusion, quality improvement programs with a set of actions focused on ATB stewardship and infection prevention in ICUs may decrease the duration of ATB treatments and the use of invasive devices with the potential to decrease HAI rates.
